# Effects of a Botanical Extract Versus Minoxidil on Hair Loss-Associated Biomarkers: An In Vitro Study

**DOI:** 10.3390/cimb48070648

**Published:** 2026-06-23

**Authors:** Gülistan Öncü, Murat Türkoğlu, Ali Türkan, Hakan Sevinç

**Affiliations:** R&D Center, Biota Laboratories, Sancaktepe, 34785 Istanbul, Turkey; goncu@biotalab.com (G.Ö.); aturkan@biotalab.com (A.T.); hsevinc@biotalab.com (H.S.)

**Keywords:** botanical extract, minoxidil, HaCaT cells, IL-6, VEGF, SRD5A2

## Abstract

Current treatment options for hair loss remain limited. Therefore, this study compared a botanical extract derived from multiple plants with the pharmaceutical agent minoxidil for topical application. The evaluated parameters included inflammatory cytokines (IL-1β, IL-6, TNF-α), growth factors (TGF-β, VEGF, KGF), and 5α-reductase type II (SRD5A2) expression in the human keratinocyte cell line HaCaT, as measured by ELISA. Both the botanical extract and minoxidil reduced IL-6 levels by 21% and 35%, and TNF-α levels by 13% and 35%, respectively. Treatment with the botanical extract and minoxidil increased VEGF expression by 50% and 85%, and KGF by 16% and 31%, respectively, while reducing SRD5A2 expression by 21% and 28%, respectively. Overall, the results of this in vitro study suggest that the botanical extract exhibits a response pattern similar to that of minoxidil, characterized by the suppression of pro-inflammatory cytokines and SRD5A2, along with enhanced expression of growth factors VEGF and KGF in HaCaT cells. These results provide a promising basis for further in vivo studies.

## 1. Introduction

Hair loss, depending on its type, is generally not a serious medical condition but can significantly impact psychological well-being, potentially causing lowered self-esteem, depression, anxiety, or social withdrawal [1].

Androgenetic alopecia (AGA) driven largely by androgens is one of the most common hair loss problems affecting both men and women [2]. Testosterone is converted to dihydrotestosterone (DHT) by the enzyme 5α-reductase type II (SRD5A2), which adversely affects the hair growth cycle [3]. In addition to DHT formation, perifollicular inflammation was also observed in AGA patients [4]. Another prevalent form of hair loss is telogen effluvium (TE), which can be associated with various chronic illnesses. There is also a link between stress and TE [5,6].

Current treatment options for hair loss remain limited. The primary pharmaceutical anti-hair loss agents include topical minoxidil, oral finasteride, and dutasteride, alongside alternative approaches such as herbal-based cosmetic products [7]. Minoxidil stands out as one of the most effective treatments and was first approved by the FDA for this purpose in 1988 [8].

The hair-growth-promoting effects of topical minoxidil are attributed to several mechanisms, including the activation of ATP-sensitive potassium channels by its active metabolite, minoxidil sulfate, as well as its vasodilatory effects that enhance cutaneous blood flow [9,10]. Additional pathways involve stimulation of growth factors such as IGF-1 and VEGF; for instance, minoxidil elevates adenosine levels in keratinocytes, which then promotes VEGF production in dermal papilla cells, thereby supporting angiogenesis [11]. In vitro studies have further demonstrated anti-inflammatory actions (e.g., reduced IL-1α expression in HaCaT cells) and increased trichohyalin expression in keratinocyte cultures [12,13,14].

Botanical extracts are widely utilized in anti-hair-loss formulations due to their high content of bioactive phytochemicals. These include secondary metabolites such as phenolic compounds, encompassing flavonoids (e.g., apigenin, chlorogenic acid, catechins, quercetin, and kaempferol), as well as tannins, phenolic acids, and coumarins. Botanical actives may mitigate hair loss by inhibiting pro-inflammatory cytokines, enhancing growth factor expression, and suppressing SRD5A2 activity [15,16,17].

In addition, phenolic compounds exhibit potent antioxidant properties, contributing to the attenuation of oxidative stress and inflammation. Several clinical studies evaluating cosmetic products containing botanical extracts for hair loss prevention have demonstrated the safety and efficacy of botanical-based shampoos and serums in the management of androgenetic alopecia (AGA) and telogen effluvium (TE). These formulations have been shown to reduce hair shedding and improve scalp health [7,18].

We previously conducted a clinical study in patients experiencing hair loss using a combination of botanical extracts derived from various plants [7], in which the extract was found to be both effective and safe. In the present study, we aimed to further investigate the effects of the same botanical extract on hair-loss-related biomarkers, comparing its activity with that of minoxidil in the human keratinocyte cell line HaCaT. The biomarkers tested were inflammatory cytokines (IL-1β, IL-6, TNF-α), growth factors (TGF-β, VEGF, KGF), and SRD5A2.

## 2. Materials and Methods

### 2.1. The Botanical Extract

The proprietary botanical extract (Biocomplex B11™) was manufactured by Martin Bauer (Vestenbergsgreuth, Germany) as a spray-dried powder derived from the following plant materials: *Urtica dioica* root, *Urtica urens* leaf, *Equisetum arvense* leaf, *Achillea millefolium* aerial parts, *Matricaria chamomilla* flowers, and *Ceratonia siliqua* fruit. The chemical profile of the extract has been previously reported and includes vitamins (B1, B2, B6, and C), minerals (iron, copper, and zinc), and flavonoids such as myricetin, quercetin, and kaempferol [19].

### 2.2. Preparation of Minoxidil and Botanical Extract

A stock solution of Minoxidil (100 mM, ICROM, Milan, Italy, The European Pharmacopoeia Quality Standard) was prepared in 50% propylene glycol, 30% ethanol, and 20% PBS. A stock solution was prepared by dissolving the botanical extract powder in PBS at a concentration of 10 mg/mL.

### 2.3. HaCaT Cells and Cell Culture

HaCaT cells are the spontaneously immortalized human keratinocyte cell line that has been used for studies of the epidermal homeostasis and its pathophysiology [20]. HaCaT cell line has a high differentiation potential in cell culture, based on the expression of various epidermal differentiation markers [21]. The HaCaT cells (ATCC, Manassas, VA, USA) were cultured in Dulbecco’s Modified Eagle Medium (DMEM; Gibco, Thermo Fisher Scientific, Waltham, MA, USA) supplemented with 10% fetal bovine serum (FBS; Sigma-Aldrich, Burlington, MA, USA), 2 mM L-glutamine (Gibco, Thermo Fisher Scientific, Waltham, MA, USA), and 1% penicillin–streptomycin (Sigma-Aldrich, Burlington, MA, USA). The cells were incubated at 37 °C in a humidified atmosphere containing 5% CO_2_. The culture medium was refreshed every three days, and the cells were passaged at 80% confluency.

### 2.4. Cell Viability Assay

The effects of minoxidil and botanical extract on HaCaT cell viability were determined by the 2,3-bis(2-methoxy-4-nitro-5-sulfophenyl)-2H-tetrazolium-5-carboxanilide (XTT) assay (Roche Diagnostics, Mannheim, Germany) following the manufacturer’s instructions. The HaCaT cells were seeded in 96-well culture plates at 10^4^ cells per well and allowed to adhere overnight. Then, the cells were treated with minoxidil at concentrations ranging from 10 to 100 µM and botanical extract at concentrations ranging from 1 µg/mL to 200 µg/mL for 24, 48 and 72 h. Vehicle control was added to check for any toxic effects of the solvent in which minoxidil was dissolved on the cells. After treatment, 50 µL of the XTT labeling mixture was added to each well and incubated for 4 h at 37 °C. Absorbance was recorded at 490 nm using 650 nm as the reference wavelength with a microplate reader (Bio-Rad, Hercules, CA, USA). Cell viability was reported as a percentage of the untreated control.

### 2.5. Treatment of HaCaT Cells with Minoxidil and Botanical Extract

The HaCaT cells were seeded at a density of 10^5^ cells/well into 6-well microplates and incubated overnight to allow cell attachment. To determine the protein levels of IL-6, IL-1β, TNF-α, VEGF, KGF, and TGF-β, the cells were treated separately with 25 μM Minoxidil and 20 μg/mL botanical extract powder for 24 h. For VEGF, TGF, and TGF- β growth factors, the cells were incubated in serum-free medium. At the end of the incubation period, cell culture supernatants were collected and centrifuged to remove cell debris. The supernatants were aliquoted and stored at −80 °C until further analysis. To determine of SRD5A2 protein levels, the cells were seeded at a density of 10^6^ cells/flask in T25 culture flasks and incubated overnight for adherence. The cells were then treated separately with 25 μM Minoxidil and 20 μg/mL botanical extract powder for 24 h. After the incubation period, the cells were collected and washed twice with cold Dulbecco’s phosphate-buffered saline (dPBS, Pan Biotech, Aidenbach, Germany). Protease inhibitor cocktail (Thermo Scientific, Waltham, MA, USA) was added to the cold PBS, and cells were lysed using the freeze–thaw method. The lysates were centrifuged at 6000× *g* for 10 min at 4 °C, and the supernatants were aliquoted and stored at −80 °C until further use for protein quantification using the Bicinchoninic Acid (BCA) assay and for subsequent enzyme-linked immunosorbent assay (ELISA) analyses.

### 2.6. Enzyme-Linked Immunosorbent Assay (Elisa)

Protein concentrations in cell lysates were determined using a BCA protein assay kit (Thermo Scientific, Waltham, MA, USA) to enable sample normalization (i.e., each sample was added at 10 µg). Levels of IL-1β, IL-6, TNF-α, VEGF, KGF, TGF-β, and SRD5A2 were measured using ELISA kits (YL Biont, Shanghai, China) according to the manufacturer’s protocols. Absorbance at 450 nm was measured using a microplate reader (Bio-Rad, Hercules, CA, USA), and all experiments were conducted in triplicate.

### 2.7. Statistical Analysis

All experiments were performed in triplicate as independent samples, and data are expressed as mean ± standard deviation (SD). Statistical differences were evaluated using Student’s *t*-test. Values of *p* < 0.05 were considered statistically significant. Data analysis was performed using OriginLab software (version 10.25).

## 3. Results

### 3.1. Concentration Optimization of Botanical Extract and Minoxidil

**Figure 1:**
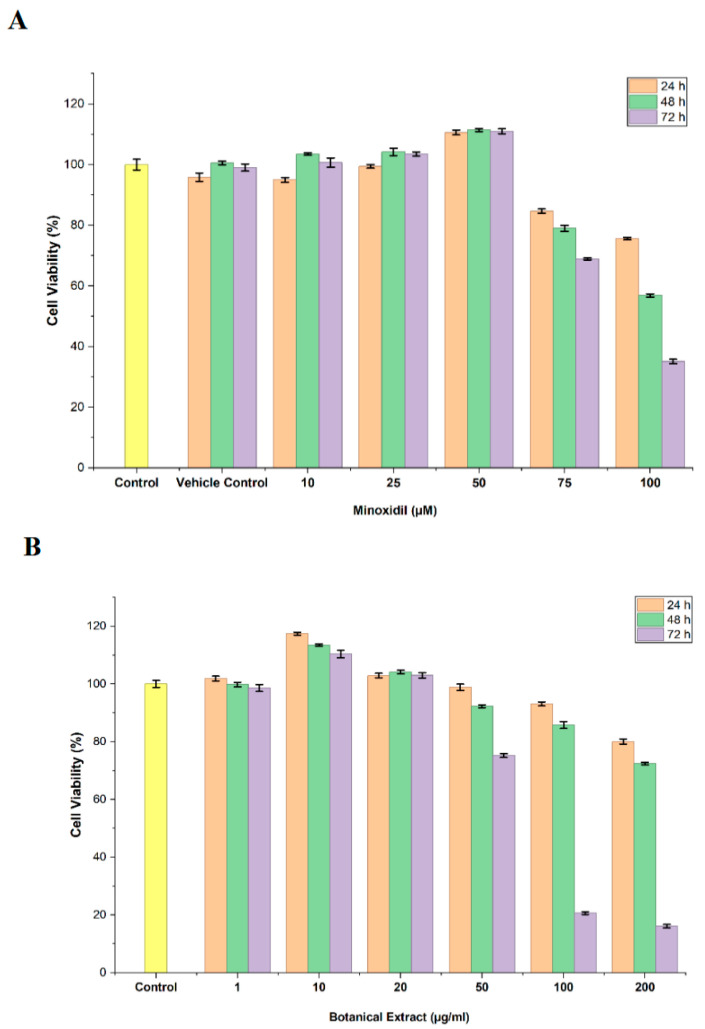
Effect of minoxidil (**A**) and botanical extract (**B**) on the cell viability of HaCaT cells. Cells were treated with increasing concentrations of minoxidil (10–100 μM) and botanical extract (1–200 μg/mL) for 24 h (orange), 48 h (green) and 72 h (purple). Control cells (yellow) were maintained without treatment. Cell viability was evaluated using the XTT assay and presented as a percentage relative to untreated controls. Data represent the mean ± SD (standard deviation) of three independent experiments.

Cell viability assays showed that concentration- and time-dependent effects of both botanical extract and minoxidil on HaCaT cells (Figure 1). Minoxidil concentrations between 10 and 50 µM did not reduce cell viability at any time point compared to the control group. Noticeable toxic effects were observed at 75 and 100 µM. Similarly, in botanical extract treatments ranging from 1 to 20 µg/mL, it was observed that high cell viability rates were maintained, and even slightly increased cell proliferation was observed in some concentrations compared to the control group. In contrast, cell viability progressively declined at 50 µg/mL, indicating the onset of mild cytotoxicity. Higher concentrations (100–200 µg/mL) significantly reduced time-dependent viability. Therefore, based on these results, concentrations of 25 µM minoxidil and 20 µg/mL botanical extract were selected for use in the subsequent studies.

### 3.2. Effect of Botanical Extract and Minoxidil on Inflammatory Factors

**Figure 2:**
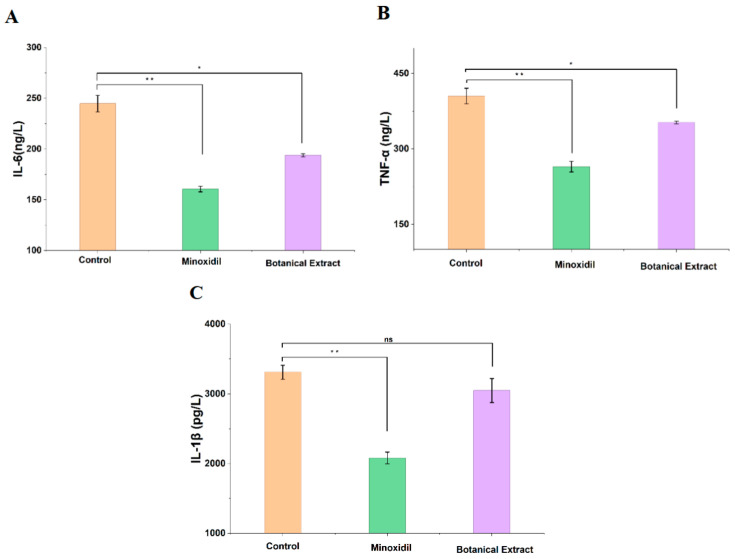
Effect of minoxidil and botanical extract treatment on the expression of inflammatory factors in HaCaT cells. The graphs show IL-6 (**A**), TNF-α (**B**), and IL-1β (**C**) levels in the control (orange) and presence of 25 μM minoxidil (green) and 20 μg/mL botanical extract (purple). Data are presented as mean ± SD (*n* = 3). Statistical comparisons were performed, and significance levels are indicated above the bars (*, *p* < 0.05; **, *p* < 0.01; ns, not significant).

In this study, the botanical extract and minoxidil exhibited similar response patterns to the evaluated factors (Figure 2). Both treatments significantly reduced levels of IL-6 (botanical extract: *p* = 0.0131; minoxidil: *p* = 0.0052) and TNF-α (botanical extract: *p* = 0.0422; minoxidil: *p* = 0.0089). In contrast, minoxidil alone produced a significant decrease in IL-1β (*p* = 0.0056), whereas the effect of the extract on this cytokine was not statistically significant. Overall, both treatments demonstrated efficacy in reducing inflammatory markers, with minoxidil showing a somewhat greater impact. The most pronounced reduction was observed for IL-6, where minoxidil achieved a 1.5-fold decrease and the extract a 1.25-fold decrease.

### 3.3. Effect of Botanical Extract and Minoxidil on Growth Factors

**Figure 3:**
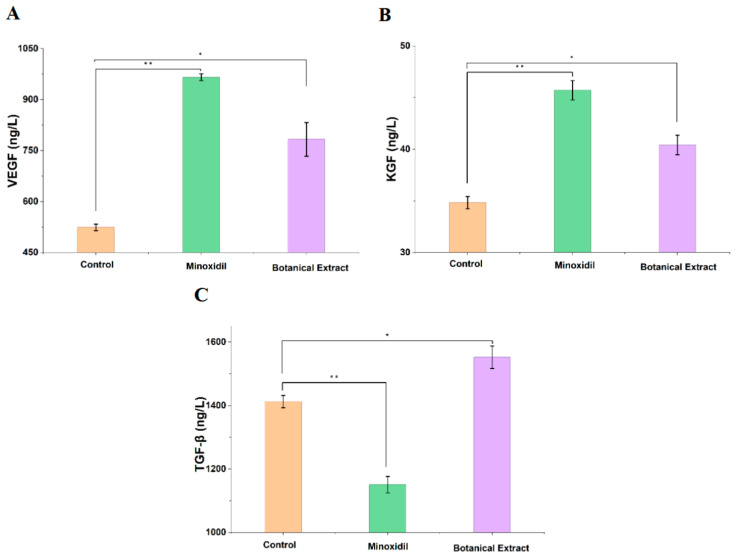
Effect of minoxidil and botanical extract treatment on the expression of growth factors in HaCaT cells. The graphs show VEGF (**A**), KGF (**B**), and TGF-β (**C**) levels in the control (orange) and presence of 25 μM minoxidil (green) and 20 μg/mL botanical extract (purple). Data are presented as mean ± SD (*n* = 3). Statistical comparisons were performed, and significance levels are indicated above the bars (*, *p* < 0.05; **, *p* < 0.01).

Minoxidil and the extract both significantly upregulated VEGF (botanical extract: *p* = 0.0194; minoxidil: *p* = 0.0055) and KGF (botanical extract: *p* = 0.0183; minoxidil: *p* = 0.0051) levels in HaCaT cell supernatants (Figure 3). Although both interventions produced statistically significant effects, minoxidil exhibited superior efficacy compared to the extract in enhancing these growth factors. In contrast, minoxidil markedly suppressed TGF-β (*p* = 0.0074)—a growth factor that negatively regulates hair follicle cycling—while the extract induced an increase in TGF-β (*p* = 0.0394). Overall, both treatments promoted cellular proliferation; however, the most pronounced effects were observed with minoxidil, which resulted in a 1.46-fold increase in VEGF and a 1.23-fold reduction in TGF-β.

### 3.4. Effect of Botanical Extract and Minoxidil on Testosterone Conversion Factor

**Figure 4:**
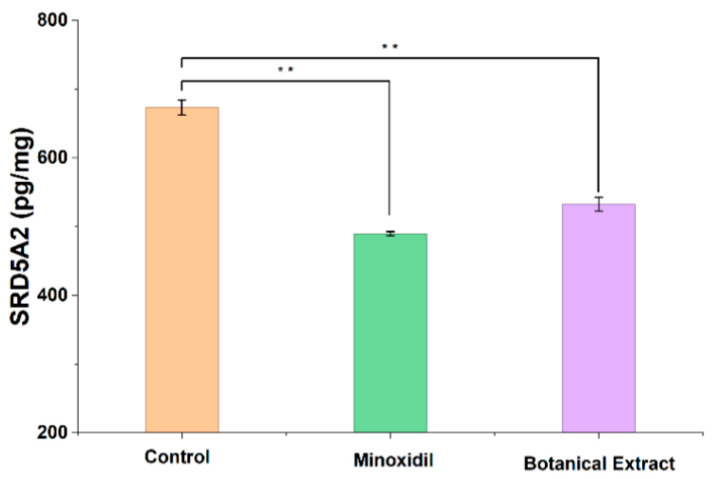
Effect of minoxidil and botanical extract treatment on the expression of testosterone conversion factor SRD5A2 in HaCaT cells. The graphs show SRD5A2 levels in the control (orange) and presence of 25 μM minoxidil (green) and 20 μg/mL botanical extract (purple). Data are presented as mean ± SD (*n* = 3). Statistical comparisons were performed, and significance levels are indicated above the bars (**, *p* < 0.01).

Both treatments were found to reduce SRD5A2 expression, consistent with our previous findings at the gene expression level [22]. *SRD5A2* gene encodes 5α-reductase type II, an enzyme responsible for converting testosterone into dihydrotestosterone (DHT), a key androgen implicated in the miniaturization of hair follicles characteristic of AGA. Interestingly, the current study demonstrates that the botanical extract, similar to minoxidil, exert a suppressive effect on SRD5A2 (botanical extract: *p* = 0.0055; minoxidil: *p* = 0.0019), suggesting a potential mechanism by which these compounds may mitigate AGA progression (Figure 4).

## 4. Discussion

The hair growth cycle, comprising the anagen, catagen, and telogen phases, is a complex process regulated by stem cells, hormones, biochemical signals, and specific enzymes. Several key factors are known to influence this cycle, including Wnt, p53, TGF-β, EGF, FGF-5, VEGF, SRD5A2, and pro-inflammatory cytokines [23]. In general, elevated levels of pro-inflammatory markers have been reported in alopecia-affected regions; notably, IL-6 levels were found to be highest in the affected scalp areas of patients with androgenetic alopecia (AGA) [24]. Additionally, IL-1α, IL-1β, and TNF-α have been identified as potent inhibitors of hair growth [25]. In the present study, we demonstrated that a flavonoid-rich botanical extract significantly reduced the protein levels of key pro-inflammatory markers, namely, IL-6 and TNF-α. In contrast, minoxidil reduced all evaluated pro-inflammatory markers. While attributing the anti-inflammatory effects of the botanical extract solely to flavonoids may be an overstatement, substantial evidence supports the well-established anti-inflammatory properties of flavonoids [26,27].

Minoxidil and the botanical extract both significantly increased VEGF and KGF levels in HaCaT cells (Figure 3). Elevated VEGF levels are known to stimulate perifollicular angiogenesis within hair follicles [28], whereas KGF serves as a critical mediator of follicular growth, development, and differentiation [29]. Notably, perifollicular angiogenesis is primarily associated with upregulated VEGF expression in outer root sheath keratinocytes rather than in dermal papilla cells [30]. Clinical evidence further underscores the importance of these growth factors. In a study comparing patients with telogen effluvium (TE) to healthy controls, VEGF levels were more than twofold lower in TE patients, while KGF levels were also significantly reduced in this group [31]. Moreover, a recent 56-day clinical study involving 60 subjects demonstrated that a formulation containing a botanical extract in combination with growth factors, including FGF and IGF, provided significant benefits in reducing hair loss [32]. The upregulation of VEGF and KGF (FGF-7) in hair follicle cells by various flavonoid containing-plant extracts has been reported previously [30,33].

Our study also showed that minoxidil suppressed TGF-β levels, whereas the botanical extract increased it. Many plant extracts have been reported to downregulate the expression of TGF-β1 and TGF-β2 in both cell and animal models [34]. However, *Cinnamomum osmophloeum* extract represents an exception, as it has been shown to increase TGF-β2 expression [35]. TGF-β is generally considered to negatively affect hair growth by suppressing hair follicle function and promoting the progression of the hair cycle into the telogen phase [17]. Conversely, the inhibition of TGF-β2 signaling at either the ligand or receptor level has been reported to significantly impair hair folliculogenesis and maturation, highlighting its complex and context-dependent role [35].

Both treatments were found to reduce SRD5A2 protein expression, consistent with our previous findings at the gene expression level [29]. Interestingly, the current study demonstrates that the botanical extract, similar to minoxidil, exerts a suppressive effect on SRD5A2, suggesting a potential mechanism by which these compounds may mitigate AGA progression. The parallel behavior observed between minoxidil and the botanical extract in downregulating SRD5A2 highlights a shared therapeutic target and warrants further investigation.

## 5. Conclusions

Overall, these findings indicate that the botanical extract demonstrates a response profile comparable to minoxidil, marked by reduced levels of pro-inflammatory cytokines and SRD5A2, as well as increased expression of the growth factors KGF and VEGF in HaCaT cells. Together, these results offer a strong rationale for pursuing further in vivo studies.

## Data Availability

The original contributions presented in this study are included in the article. Further inquiries can be directed to the corresponding author.
